# Arrhythmic Risk Stratification in Patients with Arrhythmogenic Cardiomyopathy

**DOI:** 10.3390/diagnostics15091149

**Published:** 2025-04-30

**Authors:** Marisa Varrenti, Eleonora Bonvicini, Leandro Fabrizio Milillo, Ilaria Garofani, Marco Carbonaro, Matteo Baroni, Lorenzo Gigli, Giulia Colombo, Federica Giordano, Raffaele Falco, Antonio Frontera, Roberto Menè, Alberto Preda, Sara Vargiu, Patrizio Mazzone, Fabrizio Guarracini

**Affiliations:** 1 Electrophysiology Unit, De Gasperis Cardio Center, Niguarda Hospital, 20162 Milan, Italy; leandrofabrizio.milillo@ospedaleniguarda.it (L.F.M.); ilaria.garofani@ospedaleniguarda.it (I.G.); marco.carbonaro@ospedaleniguarda.it (M.C.); matteo.baroni@ospedaleniguarda.it (M.B.); lorenzo.gigli@ospedaleniguarda.it (L.G.); giulia.colombo@ospedaleniguarda.it (G.C.); federica.giordano@ospedaleniguarda.it (F.G.); raffaele.falco@ospedaleniguarda.it (R.F.); antonio.frontera@ospedaleniguarda.it (A.F.); roberto.mene@ospedaleniguarda.it (R.M.); alberto.preda@ospedaleniguarda.it (A.P.); sara.vargiu@ospedaleniguarda.it (S.V.); patrizio.mazzone@ospedaleniguarda.it (P.M.); fabrizio.guarracini@ospedaleniguarda.it (F.G.); 2 Department of Cardiology, Santa Chiara Hospital, 38122 Trento, Italy; eleonorabonvicini@yahoo.it

**Keywords:** arrhythmic risk stratification, arrhythmogenic cardiomyopathy, sudden death, cardiac arrhythmias, genetics

## Abstract

Arrhythmogenic cardiomyopathy is a heart disease in which the heart muscle is replaced by scar tissue. This is the main substrate for the development of malignant ventricular arrhythmias. Sudden cardiac death is the most common manifestation and can often be the first sign of the disease, especially in young people. Correct stratification of arrhythmic risk is essential for the management of these patients but remains a challenge for the clinical cardiologist. In this context, the aim of our work was to review the literature and to analyse the most important studies and new developments with regard to the stratification of the risk of arrhythmia in patients suffering from arrhythmogenic cardiopathy.

## 1. Introduction

Arrhythmogenic cardiomyopathy (ACM) is a myocardial disease in which the heart muscle is replaced by “non-ischemic” fibrous or fibrofatty scar tissue. Initially described as an exclusive right ventricular disease [1,2,3,4], recent updates have demonstrated not only biventricular involvement but often exclusively left ventricular involvement [4,5]. The underlying cause of ACM is due to genetic defects in the cardiac desmosomes [5], leading to progressive myocardial atrophy, usually from the epicardium towards the endocardium, with a reduction in cardiac muscle. These myocardial wall abnormalities are the substrate for the development of malignant ventricular arrhythmias and sudden cardiac death (SCD), which is the most common manifestation of ACM.

For the diagnosis of arrhythmogenic heart disease, the first criteria were proposed in 1994 and then revised by a Task Force in 2010 [4]. Although they had high diagnostic accuracy in right ventricular forms, the advent of magnetic resonance imaging and new genetic evidence required an update of these criteria, which was proposed by a team of international experts in 2020 in what is known as the Padua Criteria [4] (see Table 1).

The 2020 Padua Criteria [4] have been essential in creating a predefined pathway for the diagnosis of the disease, but until now, missed diagnoses and misdiagnoses have been common [6]. The true prevalence of ACM has been estimated at 1:1000 [7].

However, it must be taken into account that the disease is difficult to detect in the subclinical phase and that the index presentation is often ventricular arrhythmia or, worse, sudden cardiac death, especially in young people. The incidence of a first life-threatening episode of ventricular arrhythmia at 5 years is around 20% [8,9] and ACM is the most common cause of SCD in athletes and young people [10].

Prevention of sudden cardiac death through lifestyle changes, antiarrhythmic drugs, catheter ablation, and implantable cardioverter defibrillator (ICD) therapy is the cornerstone of the management of ACM. Correct arrhythmic risk stratification is essential in the management of patients with ACM but remains a challenge for the clinical cardiologist [11].

In this context, our review aims to analyse the most relevant studies on arrhythmic risk stratification in ACM to identify the most appropriate predictors of arrhythmic risk.

## 2. Medical History

Spatial and temporal differences in the incidence of arrhythmic events in the ACM population have been demonstrated. A retrospective cohort study of North American and European patients [12] reported that more ICDs were implanted for primary prevention in North America and that higher rates of sustained ventricular arrhythmias (VAs) occurred in unprotected patients. While different lifestyles and sports regulations could explain the former observation, the privatised US healthcare system could justify the rate of VA in unprotected patients, and a different approach to ICD implantation decision making cannot be excluded. As far as the temporal distribution is concerned, arrhythmic events in ACM are more frequent in the afternoon, around 6 pm, and in winter [13]. In the general population, ventricular arrhythmias and sudden cardiac death are usually associated with acute coronary syndromes and therefore have a morning peak, coinciding with increased sympathetic activity [14]. In ACM, however, the adrenergic stimulation that triggers the arrhythmic substrate is related to physical activity, which tends to be more frequent in the latter part of the day. Genetic alteration of the circadian rhythm has also been suggested as an explanation for this anomaly. As for the seasonal distribution of events, environmental and routine factors have been proposed as explanations, but it is more likely that the reason for this seasonality is based on the increased rate of infections in winter, linked to the inflammatory role that underlies this disease [15].

As also demonstrated in the study by Castelletti et al., a higher frequency of arrhythmic events in patients with arrhythmogenic heart disease was observed during the winter months, explaining the phenomenon with a higher incidence of infections during this period. The authors report that autopsy studies showed that 70% of patients had focal lymphocytic myocarditis. Supporting this hypothesis are several studies that report cases of patients mimicking acute myocarditis as active phases of arrhythmogenic heart disease, defined as “hot phases”. In this context, it is not difficult to understand why there is a higher frequency of arrhythmic events in winter in patients with arrhythmogenic heart disease, when the possible triggers of inflammation are more frequent [1,13,16]. Regarding other cardiomyopathies, inflammatory pathogenesis does not seem to be associated with an increased risk of cardiac arrhythmias in patients with hypertrophic cardiomyopathy, unlike arrhythmogenic cardiomyopathy and dilated cardiomyopathy. In fact, several studies support the idea that dilated cardiomyopathy may also have an autoimmune pathogenesis that warrants specific treatment [15].

The prevalence of ACM is higher in the second and fifth decades of life, but a worse prognosis has been associated with the early onset of the disease [17,18]. Inciardi et al. [19] compared two groups of patients with ACM: the first with a diagnosis of ACM between 17 and 68 years of age, the second with a diagnosis of ACM but who died at the maximum age of 43 years. In a similar study, Link et al. [20] showed that young age independently predicted an appropriate ICD shock or death in ICD carriers in primary prevention. A genetic predisposition could explain this association, and proper genetic screening seems to be the only way to identify these patients because the onset of symptoms was short before the arrhythmic events or even no symptoms were noted [21].

Cardiac symptoms such as dizziness, palpitations, chest pain, and shortness of breath are common in ACM patients prior to arrhythmic manifestation. In a cohort study by Rootwelt-Norberg et al. [22], 92% of patients with ACM were symptomatic before the first arrhythmic event understood as ventricular arrhythmia: in most cases (57%), there were palpitations, syncope (23%), presyncope (27%), and exercise-induced syncope (5%). A similar study by Delgado-Vega et al. analysed data from ACM patients with sudden death [23] and reported that in 68% of cases, patients presented symptoms such as syncope and presyncope in the six months prior to cardiac arrest.

Similarly, Gupta et al. [24] described cardiac symptoms observed in 41% of patients with ACM and cardiac arrest without specifying the timing. The differences in these results are probably due to the population analysed and the target event (ventricular arrhythmia in the first case, SCD in the second, and cardiac arrest in the third).

**Figure 1:**
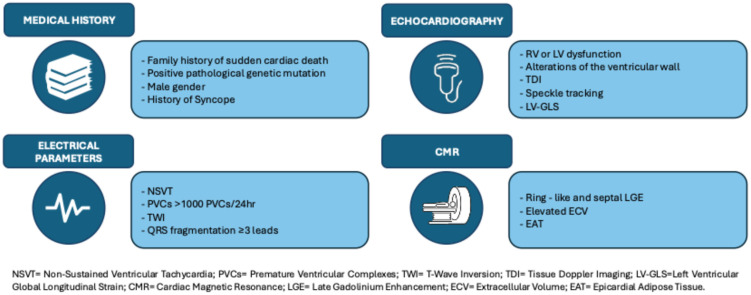
Predictors of arrhythmic risk in ACM patients. The stratification of arrhythmic risk in patients with arrhythmogenic heart disease must involve a multiparametric approach. The figure shows the main risk factors associated with an increased risk of developing life-threatening arrhythmias in various areas, ranging from clinical history to diagnostic investigations.

The strongest association between symptoms and arrhythmic events was highlighted for exercise-induced syncope, but even more specific symptoms such as dizziness and pre-syncope were present before the arrhythmic manifestation. These results suggest the importance of a detailed analysis of symptoms for correct risk stratification, and it should be considered that an earlier age of symptom onset implies a shorter time to arrhythmic manifestation (Figure 1) [24].

Beyond initial predisposition, beta-blockers are the only antiarrhythmic drugs that have been associated with a lower rate of ventricular arrhythmias. In the study by Cappelleto et al. [25], beta-blockers titrated to >50% of the target dose were shown to reduce the risk of sudden cardiac death and recurrence of monomorphic ventricular arrhythmias.

### Sex Differences

Several studies have shown that arrhythmogenic right ventricular cardiomyopathy (ARVC) predominantly affects men, who have a higher incidence of serious ventricular arrhythmias (VT/VF) and sudden cardiac death (SCD).

A Japanese study investigated sex differences in life-threatening ventricular arrhythmias and heart failure in sporadic ARVC cases. The study included 110 patients diagnosed with ARVC according to the 2010 revised Task Force criteria. Male patients accounted for 75% of the cohort. Comprehensive clinical evaluations included personal and family history, physical examination, electrocardiogram, 24 h Holter monitoring, imaging studies (transthoracic echocardiography and CMR), and electrophysiological study and myocardial biopsy, which were performed in 86% and 65% of patients, respectively. The mean follow-up was 10 years.

The study highlighted three main findings: (1) the predominance of ARVC in men, (2) a higher risk of ventricular arrhythmic events in men compared with women, and (3) a higher incidence of heart failure-related death or heart transplantation in women.

The predominance of heart failure in women with ARVC may be due to an imbalance between right ventricular volume and thoracic dimension, as hypothesised by the authors. In the advanced stages of the disease, diastolic filling of enlarged and dysfunctional RVs may be more difficult in women due to the smaller thoracic size. Conversely, the increased arrhythmic risk in men is influenced by several factors, including the effects of sex hormones, sex differences in arrhythmic substrate distribution and instability, and higher levels of vigorous physical activity in men [26].

Research into the role of sex hormones in the pathogenesis of ARVC has revealed significant gender differences. Male ARVC patients have higher testosterone levels than healthy men, whereas no significant differences in testosterone levels are observed between female ARVC patients and healthy women. Furthermore, no association was found between testosterone levels and structural or functional cardiac abnormalities at baseline or during follow-up. According to Ren J and colleagues, elevated plasma testosterone is an important predictive biomarker for adverse arrhythmic events in ARVC patients [27].

Akdis et al. further investigated the role of sex hormones in ARVC using in vitro and in vivo models. Using an induced pluripotent stem cell-derived ARVC cardiomyocyte model, their study demonstrated that testosterone exacerbates apoptosis and lipogenesis in diseased cardiomyocytes, whereas estradiol attenuates these processes. In vivo, analyses by the same authors showed that elevated testosterone levels were significantly associated with major arrhythmic events. Low levels of estradiol and dehydroepiandrosterone sulphate (DHEA-S) were associated with increased ventricular arrhythmias. In addition, elevated high-sensitivity troponin T (hs-TnT) and pro-brain natriuretic peptide (pro-BNP) levels were also correlated with increased arrhythmic events, reflecting advanced disease stages and ongoing myocardial injury during “hot phases” of ARVC characterised by increased arrhythmic activity. These findings highlight the importance of assessing sex hormone levels for arrhythmic risk stratification in AVRC [28]. However, there is no evidence regarding possible therapeutic strategies to mitigate the hormonal effect on arrhythmic risk. Future studies investigating this aspect will be important.

Sex differences in arrhythmic substrate localisation, distribution, and electrical instability have also been documented. A Taiwanese study analysed ARVC patients with drug-refractory ventricular arrhythmias (VAs) undergoing radiofrequency catheter ablation (RFCA). Electroanatomic mapping revealed that males had larger epicardial RV unipolar low voltage zones, larger areas of late potentials in both the endocardium and epicardium, and prolonged local abnormal ventricular activity. Male sex and the presence of an extensive endocardial late potential zone were identified as independent predictors of arrhythmic recurrence after ablation [29].

Differential clinical manifestations of ARVC between the sexes may also be partly explained by physical activity, a well-established exacerbating factor in ARVC. In a longitudinal cohort study of 190 ARVC patients, men had a higher incidence of ventricular arrhythmias and a greater amount of physical activity, measured in metabolic equivalents of task (MET)-hours/week. When these data were subsequently adjusted for exercise dose, no significant differences in the incidence of arrhythmic events were observed between the sexes. These findings suggest that exercise is a critical determinant of arrhythmic risk in ARVC men, and women should not be considered at lower risk based on gender. Interestingly, both sexes showed similar disease progression in terms of RV, LV, or biventricular enlargement and functional decline. In addition, a reduced tendency for disease progression was observed when exercise restrictions were implemented [30].

## 3. Electrical Parameters

The 2015 International Task Force Consensus Statement for the Treatment of Arrhythmogenic Right Ventricular Cardiomyopathy/Dysplasia [31] identified non-sustained ventricular tachycardia (NSVT) as a “major” risk factor to warrant ICD implantation in this population, while in 2011, Bhonsale et al. [32] recognised that a burden of premature ventricular contractions (PVCs) > 1000 PVCs/24 h was associated with an increased risk of ICD therapy. From these and other studies, Bosman et al. [33] confirmed in the 2022 meta-analysis that PVCs > 1000 PVCs/24 h and previous (non-)sustained ventricular tachycardia and ventricular fibrillation consistently predicted ventricular arrhythmias in ACM patients, laying the foundation for the current 2023 ESC guidelines.

In addition to the burden of ventricular arrhythmias, correct interpretation of the electrocardiogram (ECG) has been shown to help predict arrhythmic risk in ACM patients. Alteration of repolarisation due to myocardial replacement is the most common finding on the ACM ECG. The prolongation of negative T-waves on the 12-lead ECG has been shown to provide a non-invasive estimate of the amount of scar tissue and to be a predictor of arrhythmic events [34].

In a longitudinal multicentre study of 353 patients [35], T-wave inversion (TWI) in lead V3 [HR 2.03; *p* = 0.006] was associated with VA during follow-up, independent of age, sex or subject status, whereas a weaker correlation with TWI in precordial leads > V3 was demonstrated. Other studies show that T-wave inversion beyond lead V3 is associated with a more severe reduction in left ventricular systolic function but not with ventricular arrhythmic events ADDIN EN.CITE [36].

T-wave inversion in the inferior leads was also recorded, but more advanced disease was present, and only aVF [HR 1.87; *p* = 0.016] was associated with VA during follow-up, independent of age, sex, or subject status [35].

While other previous works support what is described in this recent study [9,36], it is interesting to note the result of a cohort study [37] of 278 patients, which suggested that patients with arrhythmic events had a flattened or positive T-wave in the aVR, associated with alterations in electrical activity, particularly in the right ventricular outflow tract.

In this context, the use of T-wave alternans (TWAs) as a risk predictor has been evaluated; TWAs is a calculated value that represents the spatial or temporal variations in ventricular repolarisation and has been validated to quantify arrhythmic risk in other diseases such as heart failure [38]. With regard to ACM, Chung et al. [39] proposed a cut-off value of 66 mV for TWAs as a predictor of ventricular tachyarrhythmias (with a sensitivity and a specificity of 89.5% and 90.5%, respectively). However, additional studies are needed to validate this parameter as a predictor of arrhythmic events in patients with ACM.

Similarly, inhomogeneity of myocardial electrical activity due to myocardial scar has been shown to be represented by QRS fragmentation and dispersion. In ACM, several studies reported that QRS fragmentation was significantly associated with arrhythmic events [36,40,41,42]; Canpolat et al. [41] identified QRS fragmentation ≥ 3 leads as a cut-off for patients with arrhythmic events (sensitivity 56.0%; specificity 99.7%). Less evidence has been reported for QRS dispersion [43], QRS amplitude ratio [36], and QT dispersion [43] as a predictor of arrhythmic events (Figure 1).

With regard to Holter ECG analysis, an association between arrhythmic events and impaired HRV has also been suggested [44,45]. Impaired HRV represents an imbalance of the sympatho-vagal system with reduced vagal tone and/or increased sympathetic influence, which appears to be responsible for an increased rate of ventricular arrhythmias and sudden cardiac death.

Electrophysiological studies have been carried out in ACM, but there are little data available, and they should always be considered as second-level investigations. In the study by Wang et al. conducted on 131 patients with ARVC, they report that, in univariate analysis, inducibility on electrophysiology study was a predictive factor for major arrhythmic events (unadjusted HR, (95% CI), 6. 1 (1.8–21.1)), although only cardiac syncope and probation status remained significant in the multivariate analysis [46]. Migliore et al. also report that the electrophysiological study had a positive predictive value of only 32% and that a quarter of patients with a negative study experienced an event during follow-up. However, the extent of bipolar RV endocardial low voltage areas has been reported as a strong predictor of arrhythmic outcomes in ACM [47]. The latest European guidelines on the prevention of sudden cardiac death recommend electrophysiological study in patients with SCA and symptoms suggestive of ventricular arrhythmias to define arrhythmic risk stratification; moreover, the guidelines also recommend ICD implantation in symptomatic patients with moderate right ventricular dysfunction (<40%) and/or left ventricular dysfunction (<45%) with a positive electrophysiological study [48].

## 4. Diagnostics

Arrhythmic risk stratification is a critical step in the clinical management of patients with ACM to identify those at the highest risk and to plan preventive interventions, such as implantable cardioverter defibrillator (ICD) implantation.

Among the diagnostic tools, echocardiography and cardiac magnetic resonance (CMR) play a central role due to their diagnostic and prognostic value. Information derived from both techniques has been incorporated into the updated European Task Force criteria for diagnosing ACM [49].

### 4.1. Echocardiography

Specific echocardiographic parameters have been identified as important predictors of arrhythmic risk in numerous studies over the years. Right ventricular (RV) or left ventricular (LV) dilatation, identified by two-dimensional measurements, is a marker of disease progression and structural dysfunction associated with arrhythmic risk. Several studies have shown an association between increased LV end-diastolic (LVED) and RV end-diastolic (RVED) volumes and cardiovascular or arrhythmic events in patients with ARVD.

In their study, Leren et al. demonstrated that patients with ACM and ventricular arrhythmias had a larger right ventricular diameter (40 ± 4 mm vs. 37 ± 5 mm) and more pronounced Right Ventricular Mechanical Dispersion (RVMD) (39 ± 15 ms vs. 26 ± 11 ms) compared to patients without ventricular arrhythmic events (all *p* ≤ 0.05) [50].

Global or regional ventricular dysfunction, particularly in the RV but also in the LV, is a significant predictor of arrhythmic events [49]. A reduced EF, especially in the RV, strongly correlates with arrhythmic events as it reflects advanced structural and electrical remodelling [51]. LV involvement, particularly in biventricular or LV-dominant phenotypes, is increasingly recognised and associated with worse outcomes [52].

Abnormalities such as hypokinesia, akinesia, or dyskinesia suggest localised structural damage, predisposing these regions to arrhythmogenesis. Dyskinetic or aneurysmal segments detected via echocardiography or CMR correlate strongly with ventricular arrhythmias, particularly in the presence of fibrofatty replacement [49].

The RV plays a central role in ACM, making its functional evaluation essential for risk stratification. Among various parameters, TAPSE is a simple yet informative echocardiographic marker of RV function [50,53].

Fractional Area Change (FAC), RV free wall strain, and RVOT function, derived from echocardiography or CMR, have also gained attention for arrhythmic risk stratification [53].

Tissue Doppler imaging (TDI) provides quantitative information on ventricular function. A prospective study of 72 patients with ARVD showed that tricuspid, septal, and lateral TDI S′ values were significantly lower in patients with cardiovascular death or heart transplantation. In addition, tricuspid and septal TDI S′ values were associated with an increased risk of sustained ventricular arrhythmias, highlighting the utility of TDI in the early stages of the disease [53].

Speckle tracking techniques allow the detection of subclinical myocardial dysfunction and are particularly useful in the early stages of the disease. Increased Right Ventricular Mechanical Dispersion (RVMD) and larger RV diameters correlate with ventricular arrhythmias (VAs) in early-stage ACM [50].

A 2023 study of 150 ARVC patients followed for six years found that RV deformation patterns and RV free wall longitudinal strain (RVFWLS) were the strongest independent predictors of first sustained VA [54]. When combined with the ARVC risk calculator [55], these indices significantly enhanced risk prediction, particularly for intermediate-risk patients (5–25% at five years). This approach could guide ICD implantation timing, as patients with normal RV deformation patterns at baseline showed no VA events within five years.

Normal RV deformation in the sub-tricuspid region has been associated with a lack of disease progression in relatives of ARVC patients, which may aid in family screening protocols and optimisation of follow-up [56].

Left Ventricular Global Longitudinal Strain (LV-GLS), especially epicardial GLS, has emerged as an important marker of arrhythmic risk. Studies suggest that epicardial GLS correlates with the presence of NSVT or late gadolinium enhancement (LGE). A cut-off value of approximately −16% to −15% may help identify individuals at higher risk of VA or sudden cardiac death (SCD). The addition of GLS to routine echocardiography improves the ability to identify high-risk patients [57] (Figure 1).

Layer Specific Strain (LSS) echocardiography is a promising non-invasive tool that is also for identifying early myocardial remodelling and fibrosis in Left Ventricular Arrhythmogenic Cardiomyopathy (LVAC), particularly when CMR availability is limited. A recent study, in fact, shows that LSS can act as a surrogate marker for detecting LGE and early LV fibrosis, particularly in individuals with no apparent LV dysfunction, even if it does not offer incremental diagnostic value over CMR for LGE localisation [58].

### 4.2. Cardiac Magnetic Resonance

Cardiovascular magnetic resonance imaging (CMRI) has become a pivotal non-invasive diagnostic tool for arrhythmogenic right ventricular cardiomyopathy (ARVC). This advanced imaging modality provides a detailed assessment of cardiac morphology, function, and histological changes, providing valuable insight into the location and extent of myocardial damage in vivo. A distinctive feature of CMRI is its capacity to detect intramyocardial fibrosis and fatty replacement, which can precede functional abnormalities, thereby enabling earlier disease detection.

Key parameters evaluated by CMRI in ARVC include late gadolinium enhancement (LGE) distribution, right and left ventricular function, and T1–T2 mapping sequences [59,60,61].

Studies have demonstrated the prognostic value of LGE in predicting ventricular arrhythmias (VAs) and sudden cardiac death (SCD) [59,60,61,62,63].

The presence and location of LGE serve as predictive markers of arrhythmic risk, as shown by multicentre research on ARVC patients with “ring-like” scars, which revealed a significantly higher incidence of arrhythmic events in these individuals, particularly when associated with QRS enlargement and increased LVEDVi [64]. Further research highlights the association of LGE distribution with arrhythmic outcomes. For instance, a study on ARVC patients with biventricular or left ventricular (LV) involvement found that sustained VAs occurred more frequently in those with a ring-like LGE pattern than in other LGE distributions. This finding underscores the high arrhythmic risk linked to this specific scar pattern [65]. Another study demonstrated that septal LGE was linked to a significantly higher risk of sudden cardiac death (SCD) or major ventricular arrhythmias [63].

T1 mapping has emerged as a promising technique for detecting diffuse myocardial fibrosis, a potential substrate for re-entrant VA mechanisms in ARVC [59,66].

In a study by Lu et al., elevated extracellular volume (ECV) on T1 mapping was strongly correlated with higher arrhythmic risk during follow-up. Specifically, a 1% increase in ECV was associated with a 13% rise in the risk of sustained VA after adjusting for the ARVC risk score [66]. Furthermore, CMRI feature tracking (FT) has demonstrated potential in the early detection of ventricular function changes prior to a decline in ejection fraction (EF), thereby contributing to the stratification of arrhythmic risk [66].

Cardiac magnetic resonance imaging with feature tracking (CMR-FT) has been shown to offer superior spatial resolution and less operator dependence than echocardiographic techniques such as speckle tracking echocardiography (STE). A study conducted by Augustine et al. compared CMR-FT and STE [67], demonstrating that CMR-FT provides more reproducible and accurate strain measurements, especially in patients with suboptimal echocardiographic windows due to body habitus or pulmonary interference. The higher spatial resolution of CMR allows for clearer delineation of myocardial boundaries, reducing variability due to user input or image quality. The limitations of CMR FT include higher costs compared to echocardiography and limited access to specialised centres and facilities. In addition, scanning times and post-processing analysis are longer. Although less operator-dependent in image acquisition, CMR analysis still requires qualified personnel and specialised software.

Research by Lu et al. demonstrated that each 1% worsening in LV Global Longitudinal Strain (GLS) and RV GLS increased the risk of sustained VA by 14% and 9%, respectively, over a median follow-up of 55 months [66]. Another study emphasised that left ventricular longitudinal dyssynchrony (LVLD) ≥ 89.15 ms, assessed via CMRI-FT, independently predicted cardiovascular and arrhythmic events, although the prognostic value of LV GLS and global circumferential strain (GCS) was less consistent in patients with advanced RV dysfunction [68].

Another interesting parameter that can be evaluated through CMRI is epicardial adipose tissue (EAT). A longitudinal study conducted in 2024 shows that patients with LVAC had significantly higher volumes of epicardial adipose tissue (EAT), paracardial adipose tissue (PaAT), pericardial adipose tissue (PeAT), and a higher fat ratio compared to those without. Moreover, EAT was identified as an independent predictor of MACEs alongside the 5-year ARVC risk score, and adding EAT volume to existing prognostic models (like the 5-year ARVC risk score) improved the ability to predict adverse outcomes [69].

The integration of CMRI with other diagnostic tools such as echocardiography and ECG monitoring is crucial for optimising its contribution to arrhythmic risk stratification (Figure 1). For instance, a study by Riele et al. demonstrated a strong association between baseline electrical abnormalities detected by ECG/Holter monitoring and structural/functional abnormalities identified via CMRI [51]. Notably, patients without electrical abnormalities at diagnosis had a very low risk of arrhythmias over a 7-year follow-up and showed no CMRI abnormalities, suggesting that both electrical and structural abnormalities are essential for arrhythmic occurrence.

Furthermore, the correlation between CMRI findings and QRS dispersion has been explored. CMRI-measured parameters, including the right ventricular outflow tract area, right ventricular end-diastolic volume, and end-systolic volume, have been demonstrated to be positively associated with QRS dispersion (≥40 ms). This is a robust independent predictor of SCD [70].

In conclusion, CMRI is a cornerstone in the diagnosis and risk stratification of ARVC. Its ability to identify structural and functional abnormalities, combined with advanced techniques such as T1 mapping and feature tracking, provides crucial prognostic insights. Furthermore, the integration of CMRI with other diagnostic modalities increases its utility in the management of arrhythmic risk in ARVC patients.

### 4.3. Cardiac Computer Tomography

Compared with cardiac magnetic resonance imaging (CMR), multidetector computed tomography (MDCT) offers higher spatial resolution, shorter acquisition times, and wider availability, with minimal image degradation and fewer safety concerns related to implanted defibrillators. Contrast-enhanced MDCT (CE-MDCT) has proven effective in detecting intra-myocardial fat, epicardial fat, right ventricular (RV) trabeculation, and RV wall bulging, providing significant diagnostic and therapeutic value in ARVC [71,72,73]. Studies have shown that MDCT can identify myocardial fibrofatty infiltration through reduced attenuation areas, correlating with low-voltage regions seen on electroanatomical mapping in ARVC patients. As a result, CE-MDCT can assist in identifying arrhythmogenic substrates and guiding ventricular tachycardia (VT) ablation procedures [73,74]. However, evaluating scarred myocardium with cardiac CT remains challenging due to the lack of standardised imaging protocols and the high doses of ionising radiation required. With advances in scanner technology and reductions in radiation exposure, myocardial tissue characterisation using CT may become a viable alternative when other imaging modalities are not suitable [72]. The use of increasingly advanced technology and the integration of artificial intelligence in this approach could be key to overcoming the current limitations of CT scanning in the study of patients with arrhythmogenic heart disease.

## 5. Ablative Treatment

The current guidelines recommend catheter ablation with an epicardial approach for ventricular arrhythmias in ARVC with persistent ventricular tachycardia (VT) or frequent appropriate implantable cardioverter defibrillator (ICD) interventions despite maximally tolerated antiarrhythmic therapy [75].

Christiansen MK and colleagues examined trends in transcatheter ablation (TCA) procedures and long-term outcomes in 435 patients enrolled in the Nordic ARVC Registry. These included 220 individuals with diagnosed ARVC and 215 mutation-carrying relatives. Approximately 20% of the ARVC patients underwent TCA for ventricular arrhythmias. None of the mutation-carrying relatives required the procedure. Thus, the mere presence of mutations did not predict ventricular arrhythmic events. Similarly, mutation carrier status was not associated with the need for ablation. After the ablation procedure, there was still a risk of arrhythmia recurrence; however, there was a significant reduction in the arrhythmia burden. In particular, younger age, antiarrhythmic polytherapy, and persistent ventricular arrhythmia inducibility after ablation were associated with adverse outcomes [76].

A multicentre Spanish study investigated the safety, post-ablation arrhythmic recurrence, and predictors of arrhythmic recurrence after first-line combined endo-epicardial substrate ablation in ARVC patients. The majority of the cohort (82.9%) presented with the “classic” right ventricular (RV) phenotype. The remaining 17.1% presented with left ventricular (LV) predominant ARVC. The combined endo-epicardial ablation approach achieved a high success rate. This was defined as the absence of ventricular arrhythmia inducibility at the end of the ablation procedure (89.7%). In addition, long-term follow-up showed a low rate of arrhythmia recurrence (26.8% over a mean follow-up of 32.2 ± 21.8 months) and predominant left ventricular involvement was the only predictor of arrhythmic recurrence. In advanced disease, endocardial involvement was the predominant arrhythmic substrate. This finding may be a guide to the choice of an exclusively endocardial ablation approach in such cases rather than a combined endo-epicardial strategy. Notably, the complications reported (5%)—all related to cardiac tamponade—were associated with epicardial puncture in patients with advanced disease [77].

The presence of right ventricular (RV) dysfunction is another factor influencing ablation outcomes. In a pivotal study, 106 ARVC patients with or without RV dysfunction were analysed. They underwent substrate mapping and ablation. The distribution of scar areas differed between the two groups. RV dysfunction was associated with more extensive arrhythmic substrate in both epicardial and endocardial regions. Patients with RV dysfunction had larger endocardial scar areas in the inferior and peritricuspid (inflow tract) regions, whereas epicardial scar was predominantly located in the inferior wall and to a lesser extent in the RV outflow tract (RVOT). Conversely, in patients with preserved right ventricular function, the arrhythmic substrate showed a greater involvement of the RVOT. The presence of a scar area in the endocardial superior tricuspid region was correlated with a higher risk of VT/ventricular fibrillation (VF) recurrence after ablation [78].

In addition to RV dysfunction, biventricular involvement affects the outcome of ablation. The biventricular phenotype is associated with a more extensive arrhythmic substrate, a faster rate of ventricular arrhythmias, and a higher rate of inducibility. Moreover, left ventricular VTs (LV-VTs) are more frequently associated with biventricular involvement and reduced left ventricular (LV) function. These LV-VTs are correlated with higher rates of acute failure during catheter ablation [79].

In summary, the primary predictors of arrhythmic recurrence after TCA in ARVC include younger age at disease onset, RV dysfunction, predominant left or biventricular involvement, scar area in the endocardial superior tricuspid region, use of multiple antiarrhythmic drugs, and incomplete acute ablation success as evidenced by persistent ventricular arrhythmia inducibility.

## 6. Risk Score

In the context of exploring potential predictors of arrhythmic events in patients with arrhythmogenic heart disease, Peters et al. demonstrated that right ventricular dilatation and ECG repolarisation abnormalities (e.g., right bundle branch block and T-wave inversions in precordial leads above V3) were identified as significant risk factors for arrhythmic events in this patient population [80].

Conversely, it has been established that the extension of negative T-waves in the 12 leads serves as a non-invasive estimate of the extent of the underlying scar, and consequently, an augmented arrhythmic risk [34]. Furthermore, QRS fragmentation has been identified as an indirect expression of myocardial scar, correlating with an escalation in arrhythmic events in these patients [42].

**Figure 2:**
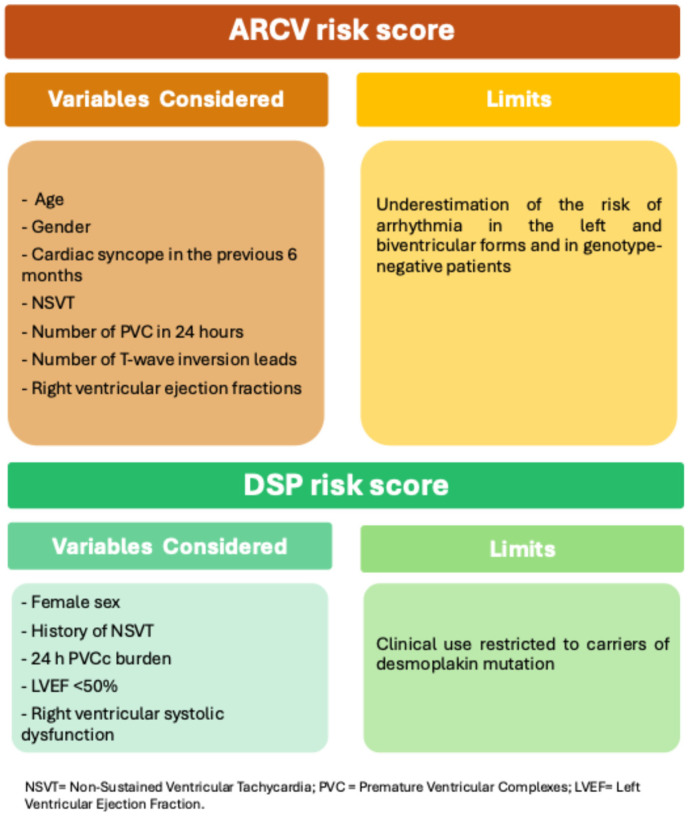
Risk Score. NSVT = non-sustained ventricular tachycardia; PVCs = premature ventricular complexes; LVEF = Left Ventricular Ejection Fraction.

In 2019, Cadrin-Tourigny J and colleagues developed a risk score predicting the 5-year risk of ventricular arrhythmic events in patients with ARVC based on the presence of seven predictors: age, gender, cardiac syncope in the previous 6 months, non-sustained ventricular tachycardia, number of premature ventricular complexes in 24 h, number of T-wave inversion leads, and right ventricular ejection fractions (ARVCrisk.com) (Figure 2) [55].

The score has been validated in other retrospective studies conducted on large cohorts of patients with ARVC, supporting the use of this score in the decision-making process for the indication of ICD implantation [81]. However, given that the patient’s clinical situation may change over time, it is necessary to update risk stratification periodically [82].

Furthermore, it should be considered how the same algorithm may underestimate the arrhythmic risk over time in left and biventricular forms and in genotype-negative forms [83,84,85].

A novel risk score predicting the likelihood of ventricular arrhythmic events in patients diagnosed with arrhythmogenic heart disease and carrying a Desmoplakin gene mutation has recently been published (Figure 2). The study identified five parameters as predictors of arrhythmic events: female sex, history of non-sustained ventricular arrhythmias, number of ventricular extrasystoles in 24 h, and presence of moderate or severe right ventricular dysfunction. The model’s discriminatory capacity has been demonstrated, suggesting that it could guide the selection of defibrillator implantation for this patient population [86].

## 7. Genetics

Genetics has been demonstrated to play a significant role in the management of patients diagnosed with arrhythmogenic cardiomyopathies, both in terms of diagnosis and in the stratification of arrhythmic risk [87]. Mutations in desmosomal genes have been observed in 50% of patients with arrhythmogenic heart disease, and these are represented by mutations in the genes for Plakophilin C (PKP2), Desmoplakin (DSP), Desmocollin-2 (DSC2), Desmoglein-2 (DSG2), and Plakoglobin (JUP) [88,89]. Furthermore, mutations in non-desmosomal genes, including Transmembrane protein 43—luma (TMEM 43), Phospholamban (PLN), Filamin C (FLNC), Desmin (DES), and LMNA (Lamin A/C), have been identified as contributing to the pathogenesis of heart disease [88] (see Table 2).

In their study of 252 patients diagnosed with arrhythmogenic right ventricular dysplasia (ARVD) and a positive genetic mutation for DSP, Gasperetti and colleagues demonstrated that 37.3% of patients experienced a ventricular arrhythmic event following a follow-up period of at least 44.5 months. The factors most associated with the occurrence of arrhythmic events at follow-up were history of TVNS, PVC burden, and left ventricular involvement [90].

While the role of genetics in the phenotyping of the disease has been firmly established, its contribution to arrhythmic risk stratification remains limited.

In 2017, Castelletti et al. conducted a study on a small cohort of DSP patients and demonstrated that they had a high arrhythmic risk [91]. Since then, subsequent studies have confirmed that DSP-related cardiomyopathy is a distinct disease with a high arrhythmic burden [90,92]. Concurrent findings have been reported for other desmosomal mutations, while a more favourable prognosis and a later onset have been demonstrated for non-desmosomal mutations [93]. In the desmosomal group, conflicting results have emerged regarding the impact of specific mutations on arrhythmic risk. In a prospective registry, Christensen et al. [94] analysed a cohort of 419 ACM patients, of whom 62% had a desmosomal gene mutation, specifically 222 patients in PKP2, 15 in DSC2, 60 in DSG2, and 26 in DSP. Multivariate analysis demonstrated a borderline significant (*p* = 0.06) higher rate of appropriate ICD therapy and electrical storm in PKP2 carriers compared to DSC2/DSG2/DSP carriers. Consistent findings have been reported in a Chinese study [7], while in European studies [93,95] the risk of sustained ventricular arrhythmias was comparable in PKP2 and other desmosomal mutations.

While a clear stratification of the arrhythmic risk for desmosomal mutation genotypes remains elusive, a multicentre retrospective observational cohort study [84] on 554 patients has shown a lower incidence of VAs and a later onset in gene-elusive patients, suggesting that the absence of known mutation could identify patients with a lower arrhythmic risk. Conversely, it has been demonstrated that carrying multiple P/LP variants of desmosomal mutations is linked with a more severe disease. Rigato et al. identified that 16% of 134 patients exhibited a complex genetic profile with multiple desmosomal mutations. Multivariable analysis revealed that this subgroup demonstrated an elevated risk of long-term major arrhythmic events and sudden cardiac death (SCD) [96].

## 8. Conclusions

Risk stratification for arrhythmia in ACM is still a challenge for clinical cardiologists. Recent advances in cardiovascular imaging techniques and the introduction of new gene-specific risk scores may help to better identify patients who are at higher risk of serious arrhythmic events during follow-up and who may benefit from defibrillator implantation for protection against sudden cardiac death. Family history of sudden cardiac death, the presence of a pathogenic genetic mutation, male sex, history of syncope, ventricular dysfunction, ventricular arrhythmic pattern, and the presence and distribution of late gadolinium enhancement on cardiac magnetic resonance imaging are the main risk factors associated with an increased likelihood of developing major ventricular arrhythmias in patients with ACM and should be carefully evaluated from the medical history to instrumental investigations. Furthermore, the clinical condition of patients is not static but dynamic over time, and periodic multiparametric reassessment is mandatory to identify any changes in clinical status and any disease progression that would require immediate intervention.

## Figures and Tables

**Table 1:** “Padua Criteria” for diagnosis of arrhythmogenic cardiomyopathy.

	Right Ventricle	Left Ventricle
**Morpho-functional ventricular abnormalities**	Major: - Regional RV akinesia, dyskinesia, or bulging plus one of the following: - Global RV dilatation (an increase in RV EDV according to the imaging test-specific nomograms); - Global RV systolic dysfunction (reduction in RV EF according to the imaging test-specific nomograms). Minor: - Regional RV akinesia, dyskinesia, or aneurysm of RV free wall.	Minor: - Global LV systolic dysfunction (depression of LV EF or reduction in echo-cardiographic global longitudinal strain), with or without LV dilatation (an increase in LV EDV according to the imaging test-specific nomograms for age, sex, and BSA). - Regional LV hypokinesia or akinesia of LV free wall, septum, or both.
**Structural myocardial abnormalities**	By CE-CMR: Major: - Transmural LGE (stria pattern) of ≥1 RV region(s) (inlet, outlet, and apex in 2 orthogonal views). By EMB (limited indications): Major: - Fibrous replacement of the myocardium in ≥1 sample, with or without fatty tissue.	Major: - LV LGE (stria pattern) of ≥1 Bull’s Eye segment(s) (in 2 orthogonal views) of the free wall (subepicardial or midmyocardial), septum, or both (excluding septal junctional LGE).
**Repolarization abnormalities**	Major: - Inverted T-waves in right precordial leads (V1, V2, and V3) or beyond in individuals with complete pubertal development (in the absence of complete RBBB). Minor: - Inverted T-waves in leads V1 and V2 in individuals with completed pubertal development (in the absence of complete RBBB). - Inverted T-waves in V1, V2, V3, and V4 in individuals with completed pubertal development in the presence of complete RBBB.	Minor: - Inverted T-waves in left precordial leads (V4-V6) (in the absence of complete LBBB).
**Depolarization abnormalities**	Minor: - Epsilon wave (reproducible low-amplitude signals between the end of the QRS complex to the onset of the T-wave) in the right precordial leads (V1 to V3). - Terminal activation duration of QRS ≥55 ms measured from the nadir of the S wave to the end of the QRS, including R’, in V1, V2, or V3 (in the absence of complete RBBB).	Minor: - Low QRS voltages (<0.5 mV peak to peak) in limb leads (in the absence of obesity, emphysema, or pericardial effusion).
**Ventricular arrhythmias**	Major: - Frequent ventricular extrasystoles (>500 per 24 h), non-sustained or sustained ventricular tachycardia of LBBB morphology. Minor: - Frequent ventricular extrasystoles (>500 per 24 h), non-sustained or sustained ventricular tachycardia of LBBB morphology with inferior axis (“RVOT pattern”).	Minor: - Frequent ventricular extrasystoles (>500 per 24 h), non-sustained or sustained ventricular tachycardia with an RBBB morphology (excluding the “fascicular pattern”).
**Family history/genetics**	Major: - ACM confirmed in a first-degree relative who meets diagnostic criteria. - ACM confirmed pathologically at autopsy or surgery in a first-degree relative. - Identification of a pathogenic or likely pathogenetic ACM mutation in the patient under evaluation. Minor: - History of ACM in a first-degree relative in whom it is not possible or practical to determine whether the family member meets diagnostic criteria. - Premature sudden death (>35 years of age) due to suspected ACM in a first-degree relative. - ACM confirmed pathologically or by diagnostic criteria in a second-degree relative.	

ACM = arrhythmogenic cardiomyopathy; BSA = body surface area; EDV = end-diastolic volume; EF = ejection fraction; LBBB = left bundle branch block; LGE = late gadolinium enhancement; LV = left ventricle; RBBB = right bundle branch block; RV = right ventricle; and RVOT = right ventricular outflow tract.

**Table 2:** Genes involved in the pathogenesis of arrhythmogenic cardiomyopathy.

Desmosomal Genes	Non-Desmosomal Genes
- Plakophilin C (PKP2) - Desmoplakin (DSP) - Desmocollin-2 (DSC2) - Desmoglein-2 (DSG2) - Plakoglobin (JUP	- Transmembrane protein 43—luma (TMEM 43) - Phospholamban (PLN) - Filamin C (FLNC) - Desmin (DES) - LMNA (Lamin A/C)

## Data Availability

Not applicable.
